# Air and environmental sampling for SARS-CoV-2 around hospitalized patients with coronavirus disease 2019 (COVID-19)

**DOI:** 10.1017/ice.2020.282

**Published:** 2020-06-08

**Authors:** Vincent Chi-Chung Cheng, Shuk-Ching Wong, Veronica Wing-Man Chan, Simon Yung-Chun So, Jonathan Hon-Kwan Chen, Cyril Chik-Yan Yip, Kwok-Hung Chan, Hin Chu, Tom Wai-Hin Chung, Siddharth Sridhar, Kelvin Kai-Wang To, Jasper Fuk-Woo Chan, Ivan Fan-Ngai Hung, Pak-Leung Ho, Kwok-Yung Yuen

**Affiliations:** 1 Department of Microbiology, Queen Mary Hospital, Hong Kong Special Administrative Region, China; 2 Infection Control Team, Queen Mary Hospital, Hong Kong West Cluster, Hong Kong Special Administrative Region, China; 3 Department of Microbiology, Li Ka Shing Faculty of Medicine, The University of Hong Kong, Hong Kong Special Administrative Region, China; 4 Department of Medicine, Li Ka Shing Faculty of Medicine, The University of Hong Kong, Hong Kong Special Administrative Region, China

## Abstract

**Background:**

The role of severe respiratory coronavirus virus 2 (SARS-CoV-2)–laden aerosols in the transmission of coronavirus disease 2019 (COVID-19) remains uncertain. Discordant findings of SARS-CoV-2 RNA in air samples were noted in early reports.

**Methods:**

Sampling of air close to 6 asymptomatic and symptomatic COVID-19 patients with and without surgical masks was performed with sampling devices using sterile gelatin filters. Frequently touched environmental surfaces near 21 patients were swabbed before daily environmental disinfection. The correlation between the viral loads of patients’ clinical samples and environmental samples was analyzed.

**Results:**

All air samples were negative for SARS-CoV-2 RNA in the 6 patients singly isolated inside airborne infection isolation rooms (AIIRs) with 12 air changes per hour. Of 377 environmental samples near 21 patients, 19 (5.0%) were positive by reverse-transcription polymerase chain reaction (RT-PCR) assay, with a median viral load of 9.2 × 10^2^ copies/mL (range, 1.1 × 10^2^ to 9.4 × 10^4^ copies/mL). The contamination rate was highest on patients’ mobile phones (6 of 77, 7.8%), followed by bed rails (4 of 74, 5.4%) and toilet door handles (4 of 76, 5.3%). We detected a significant correlation between viral load ranges in clinical samples and positivity rate of environmental samples (*P* < .001).

**Conclusion:**

SARS-CoV-2 RNA was not detectable by air samplers, which suggests that the airborne route is not the predominant mode of transmission of SARS-CoV-2. Wearing a surgical mask, appropriate hand hygiene, and thorough environmental disinfection are sufficient infection control measures for COVID-19 patients isolated singly in AIIRs. However, this conclusion may not apply during aerosol-generating procedures or in cohort wards with large numbers of COVID-19 patients.

Pandemic transmission of coronavirus disease 2019 (COVID-19) due to severe respiratory syndrome coronavirus 2 (SARS-CoV-2), a novel β-coronavirus, has caused an unprecedented public health threat in the 21st century. As of May 25, 2020, the global number of COVID-19 cases was >5.3 million, with 342,029 deaths.^1^ The rapid dissemination of disease may be attributed to the presence of asymptomatic patients with active shedding of virus,^2–5^ which may lead to direct transmission by the droplet route and indirect transmission via contact with a contaminated environment. Super-spreading events associated with explosive increase in number of cases may further complicate the transmission dynamics of COVID-19.^6,7^ The basic reproductive number of SARS-CoV-2 of (~2.7) is similar to that of SARS-CoV,^8^ both of which are markedly lower than that of airborne measles (12–18),^9^ suggesting that airborne transmission is not a major route of infection. However, clinical and experimental investigations have suggested possible airborne transmission of SARS-CoV-2.^10,11^ In the experimental setting with artificial generation of aerosol, SARS-CoV-2 remained detectable in aerosols up to 3 hours.^11^ In the clinical setting, the collection of air samples in patients’ rooms is the most direct approach to determining the presence of airborne virus, but the findings have been inconsistent.^10,12^ In a study from Singapore, SARS-CoV-2 RNA was not detected in the air samples collected adjacent to patients’ heads.^12^ A subsequent study demonstrated the presence of SARS-CoV-2 RNA in 44% of air samples in an intensive care unit (ICU) in Wuhan, China, although the average viral load in the air samples was low.^10^ Here, we performed air sampling from symptomatic and asymptomatic patients with high to low viral loads in their respiratory samples to resolve this controversy. In addition, we also conducted environmental sampling to correlate the viral load of patients’ clinical samples with the positivity rate of environmental samples. Our findings have implications for the understanding of hospital COVID-19 transmission and the formulation of infection control policy.

## Methods

### Collection of air samples inside airborne infection isolation rooms (AIIRs)

Patients with newly diagnosed COVID-19 who were hospitalized singly in AIIRs with separate toilet and shower facilities and who consented to participate in air sample collection were recruited. To increase the proportion of exhaled air sampled and to reduce the proportion of environmental air from the air conditioning system with 12 air changes per hour, an umbrella fitted with transparent plastic curtain was used as an air shelter to cover patients during sample collection (Supplementary Fig. 1a–d online). Air samples of patients inside this air shelter were collected using the Sartorius MD8 airscan sampling device (Sartorius AG, Germany) with sterile gelatin filters (80 mm in diameter and 3 µm pore size (type 17528-80-ACD) (Sartorius AG). Briefly, the air sampler was perpendicularly positioned 10 cm away from the patient’s chin. At a rate of 50 L per minute, 1,000 L of air was collected by each gelatin filter for 20 minutes while patients were with or without a surgical mask that complies with the ASTM F2100 level 1 standard. Patients 1–6 were chosen as cases to detect whether SARS-CoV-2 RNA was present in the air. As positive controls, COVID-19 patients were instructed to sneeze directly onto the gelatin filter used for the air sampler and to spit saliva droplets onto the gelatin filter. Patients 7–10 were chosen as positive controls, and patient 6 served as both a case and his own control. After air sampling or collecting the positive controls, each gelatin filter was soaked into 5 mL viral transport medium (VTM) and incubated at 37°C for 10 minutes. After dissolution of the gelatin filter, 1 mL VTM was collected for nucleic acid extraction, as we described previously.^13^

### Collection of environmental samples inside AIIRs

Swab samples from the patient’s environment, including bed rail, locker, bed table, toilet door handle, and the patient’s mobile phone, were collected for SARS-CoV-2 viral load assay before daily environmental disinfection with sodium hypochlorite solution (1,000 ppm). Briefly, swab samples covering a mean surface area of 9 cm^2^ (3 cm × 3 cm) for bed rail, locker, and bed table, and the entire surfaces of toilet door handles and patients’ mobile phones were collected. Swabs were then submerged in 2 mL VTM. Each swab sample in VTM was vortexed and centrifuged at 13,000 × *g* for 1 minute, and 1 mL of the supernatant was used for nucleic acid extraction.

The nasopharyngeal flocked swab and deep throat saliva of patients were collected on the day of environmental sampling and were subjected to viral load assay. If other clinical samples, such as throat swab, sputum, and/or rectal swab, were collected, the results were used to correlate with the results of environmental sampling. The clinical sample with the highest viral load from each patient was selected for correlation with the positivity rate of their own environmental samples.

### Virological assay

The viral load assay of air and environmental samples is illustrated in Supplementary File 1 (online). The correlation experiment between viral load and plaque-forming units was performed in triplicate. The rationale and technical details of performing this experiment are described in Supplementary File 1.

This study was approved by the Institutional Review Board of The University of Hong Kong/Hospital Authority, Hong Kong West Hospital Cluster.

### Statistical analysis

Statistical analyses were performed using the Spearman rank test using XLSTAT software (Addinsoft, New York, NY). *P* < 0.05 was considered statistically significant.

## Results

### Air samples inside AIIRs

From January 24, 2020, to April 9, 2020, clinical samples were collected from 6 hospitalized patients on the same day of their air sampling. Except for 1 asymptomatic patient with low viral load (2.07 × 10^−1^ copies/mL) in deep throat saliva collected in the early morning, the other 5 patients (patient 1 and patients 3–6) had viral loads between 3.30 × 10^6^ and 9.17 × 10^7^ copies/mL (Table 1). The results of patient 1 were reported previously.^14^ However, all of the air samples from these 6 patients collected inside the air shelter were negative for SARS-CoV-2 RNA by RT-PCR, including 1 patient (patient 6) who was put on high-flow oxygen of 100% in the ICU at the time of air sampling collection. Patients 6–10 (positive controls) were instructed to sneeze and spit saliva droplets directly onto the gelatin filters used in the air sampler. With the exception of patient 6, who had a viral load of 2.54 × 10^4^ copies/mL in the gelatin filter during sneezing, the sneezing samples onto the gelatin filters were negative for SARS-CoV-2 RNA. The saliva droplets directly spitted on gelatin filters of patients 6–10 were all positive for SARS-CoV-2 RNA (Table 1).

**Table 1.** Clinical and Epidemiological Characteristics of 6 COIVD-19 Patients (Patients 1–6) Undergoing Air Sampling, and 5 Patients (Patient 6–10) Served as Positive Control by Sneezing and Spitting^a^

Patient	1^b,c^	2^c^	3	4	5	6	7	8	9	10
Age/Sex	M/39	F/61	F/34	M/15	M/36	M/62	F/59	F/18	M/53	F/55
Source of infection	Imported (China)	Imported (Japan)	Local^d^	Local^d^	Local	Local^d^	Local^d^	Imported (UK)	Imported (Japan)	Local^d^
Day after symptom onset	D4	No	D3	D3	D4	D11	D1	D1	D4	D5
Placement of patient	AIIR (SR)	AIIR (SR)	AIIR (SR)	AIIR (SR)	AIIR (SR)	AIIR (SR)^e^	AIIR (SR)	AIIR (SR)	AIIR (SR)	AIIR (SR)
**Therapy at the day of air sample collection**										
Antiviral	No	No	No	No	No	Yes	No	No	No	No
Steroids	No	No	No	No	No	No	No	No	No	No
High-flow O_2_	No	No	No	No	No	Yes (100%)	No	No	No	No
**Covering patients with a shelter during air samples collection**^f^										
	No	Yes	Yes	Yes	Yes	Yes	NA	NA	NA	NA
**Viral load of clinical samples (copies per mL) on the day of air sample collection**										
NPS	3.30 × 10^6g^	UD	3.83 × 10^7^	6.69 × 10^8^^g^	8.40 × 10^6^	7.45 × 10^7^	2.97 × 10^8^	1.58 × 10^8^	1.07 × 10^7^	2.14 × 10^8^
DTS	5.90 × 10^6^	2.07 × 10^-1^	9.16 × 10^7^	NP	2.55 × 10^5^	1.98 × 10^7^	8.61 × 10^4^	4.71 × 10^4^	7.93 × 10^7^	1.17 × 10^6^
**Viral load of air samples (copies per mL) while wearing surgical mask**^h^										
	UD	UD	UD	UD	UD	UD	NP	NP	NP	NP
**Viral load of air samples (copies per mL) while not wearing surgical mask**										
	UD	UD	UD	UD	UD	UD	NP	NP	NP	NP
**Viral load of positive control samples (copies per mL) collected with different maneuvers**										
Sneezing directly to the gelatin filter used by the air sampler										
	NP	NP	NP	NP	NP	2.54 × 10^4^	UD	UD	UD	UD
Spitting of saliva directly onto the gelatin filter used by the air sampler										
	NP	NP	NP	NP	NP	1.07 × 10^6^	2.06 × 10^4^	1.94 × 10^4^	2.32 × 10^7^	1.00 × 10^5^

Note. AIIR, airborne infection isolation room with at least 12 air changes per hour; D, day; DTS, deep throat saliva collected in early morning before mouth wash; NA, not applicable; NP, not performed; NPS, nasopharyngeal swab; RT-PCR, reverse-transcription polymerase chain reaction; SR, single room; TS, throat swab; UD, undetectable; VL, viral load.

a Patient 1–6 were chosen as cases to detect whether SARS-CoV-2 RNA was present in the air. As positive controls, COVID-19 patients were instructed to sneeze directly and spit saliva droplets onto the gelatin filter used for the air sampler. Patients 7–10 were chosen as positive controls, while patient 6 served as both a case and his own control.

b Patient 1 was reported previously.^14^

c Air samples for SARS-CoV-2 RNA were collected by an air sampler, SAS Super ISO 180 model 86834 (VWR International PBI S.r.l., Milan, Italy).The air sampler was perpendicularly positioned at a distance of 10 cm at the patient’s chin, and 1,000 L of air at a rate of 180 L per minute was collected for each culture plate containing 3 mL of viral transport medium. Different air sampler was used for patients 3–6 and the protocol was described in the method session.

d Acquisition of SARS-CoV-2 from household member.

e Inside adult intensive care unit.

f Patients were placed under a shelter using an umbrella surrounding with a plastic curtain in order to reduce the turbulent of air flow inside the shelter.

g Pool nasopharyngeal and throat swab was taken.

h Surgical mask is ASTM (American Society of Testing and Materials) F2100 level 1 standard.

### Environmental samples inside AIIRs

**Fig. 1.**
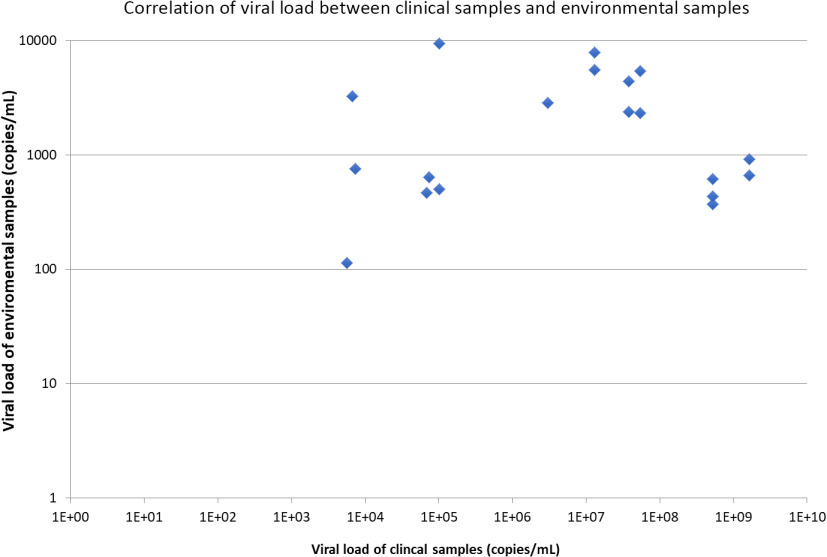
Correlation of viral load between clinical samples and environmental samples. Note. The viral load is expressed in logarithmic scale (base 10).

**Fig. 2.**
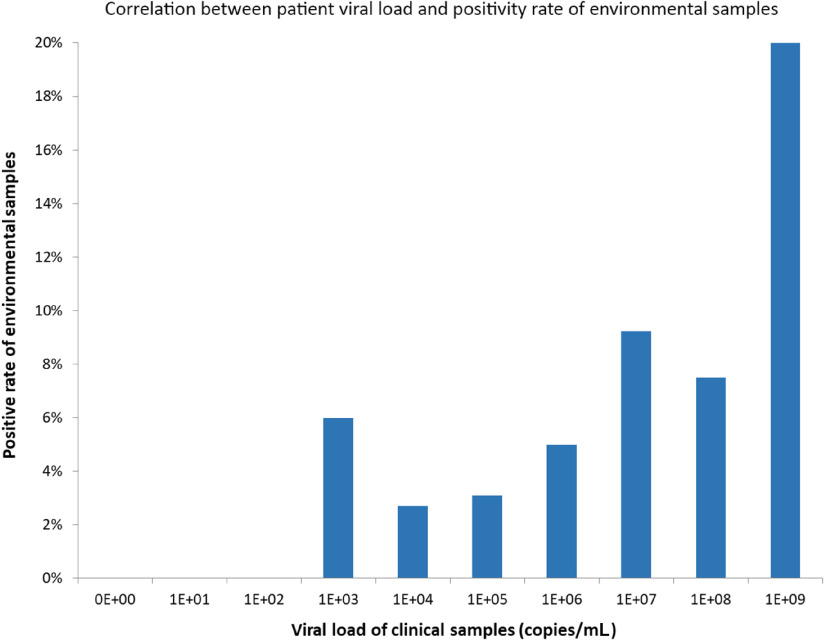
Correlation between patient viral load and positivity rate of environmental samples

Note. The clinical sample with the highest viral load from each patient was selected for correlation with the positivity rate of their own environmental samples. The numerators and denominators of environmental samples in relation to clinical samples were listed as 0/13 at 0E+00, 0/5 at 1E+01, 0/30 at 1E+02, 3/50 at 1E+03, 2/74 at 1E+04, 2/65 at 1E+05, 1/20 at 1E+06, 6/65 at 1E+07, 3/40 at 1E+08, 2/10 at 1E+09.

From February 28, 2020, to March 22, 2020, a total of 377 environmental samples from AIIRs housing 21 RT-PCR–confirmed COVID-19 patients were collected during 76 room visits before daily environmental disinfection. The median frequency of environmental sample collection per single room was 3 (range, 1–8). Of these 76 room visits for environmental sample collection, at least 1 environmental site positive for SARS-CoV-2 in 13 room visits (17.2%). In total, 19 of 377 samples (5.0%) were RT-PCR positive. Patients’ mobile phones had highest rate of contamination, followed by bed rails and toilet door handles (Table 2). Of these 19 environmental samples positive for SARS-CoV-2, the median viral load was 9.2 × 10^2^ copies/mL (range, 1.1 × 10^2^ to 9.4 × 10^4^ copies/mL). The correlation between viral loads in clinical samples of patients and environmental samples is illustrated in Figure 1, but it did not reach statistical significance (*P* = .908; Spearman ρ = −0.029). We then divided patients into groups based on viral load in clinical samples and assessed environmental contamination rates in each group (Fig. 2). Environmental sample positivity was only observed in patients with clinical samples having a viral load ≥10^3^ copies/mL. We detected a positive correlation between patient viral load (up to 9 log copies/mL) and positivity rate of environmental samples (*P* = .001; Spearman ρ = 0.090).

**Table 2.** Environmental Contamination by SARS-CoV-2 in Airborne Infection Isolation Single Room Caring COVID-19 Patients

Variable	No. of Environmental Samples Positive for SARS-CoV-2 (%)	No. of Environmental Samples Collected
Patient’s mobile phone	6 (7.8)	77
Bed rail	4 (5.4)	74
Toilet door handle (outside)	4 (5.3)	76
Bed table	3 (3.9)	76
Locker	2 (2.7)	74

Note. Environmental samples collected before the daily cleaning and disinfection of patient’s room.

### Correlation between viral load and plaque-forming units

The correlation experiment between viral load and plaque-forming units was performed in triplicate and is illustrated in Supplementary Table 1 (online). Using the RNA-dependent RNA polymerase gene as the target, 1 plaque-forming unit was equivalent to 1.67 × 10^4^ ± 4.43 × 10^3^ copies/mL.

## Discussion

In this study, we could not demonstrate the presence of SARS-CoV-2 in air samples collected 10 cm from a patient’s chin with or without a surgical mask in a single AIIR setting, even among symptomatic patients with high viral loads and in a patient receiving high-flow oxygen in the ICU. Our finding is consistent with the initial observation reported in Singapore where all air samples had no detectable SARS-CoV-2 inside the isolation room, including air samples collected when the sampler was placed next to patients’ heads.^12^ Although SARS-CoV-2 was identified in 2 of 3 air outlet fan next to patients’ heads, the level of these fans, whether at the roof or ground level, was not specified. An air outlet fan located at ground level may be easily contaminated by respiratory droplets instead of airborne aerosols. SARS-CoV-2 was detected in the air samples collected in the patients’ area in the ICU in Wuhan, China.^10^ To interpret these apparently discordant findings, we must note that there is no standardized method for collecting air samples for virological investigation.^15^ Different models of air samplers and protocols were used in our study and the other 2 previous studies. More importantly, the rates of collection and the total volumes of air collected were also different. In our study, we collected a total of 1,000 L of air at a rate of 50 L per minute for 20 minutes per sample (Table 3). The study from Singapore was conducted in a single room with negative pressure setting similar ours, and 1,200 L of air at a rate of 5 L per minute was collected, which required 4 hours per air sample collection. In the study from Wuhan, China, a total of 9,000 L of air at a rate of 300 L per minute was collected for 30 minutes per air sample within the ICU housing 15 patients, and also in a general ward housing 24 patients. The presence of SARS-CoV-2 in their air samples may be attributed to a larger volume of 9,000 L of air being collected in both the ICU and the general ward. The higher rates of positive air samples in ICUs than that in general wards may be related to the performance of aerosol-generating procedures in ICUs, especially in an ICU with an open-cubicle design. This situation is similar to the occurrence of opportunistic airborne transmission leading to the SARS outbreak in ICU in 2003.^16^

**Table 3.** Literature Review of the Clinical and Experimental Studies to Detect the Presence of Coronaviruses Including SARS-CoV-2 in Air and Environment

First Author [Reference]	Country (Year of Publication)	Setting	Patient Profile or Experimental Protocol	Detail of Air Sampling	Gene Target for RT-PCR	SARS-CoV-2 RNA in Air Samples	SARS-CoV-2 RNA in Environmental Samples
Ong SWX [12]	Singapore (2020)	Clinical; AIIR (12 ACH)	4 patients; CT value (clinical samples): 25.69–35.33	SKC Universal pumps: collected 1,200 L at 5 L/min (AIIR & anteroom)	RdRp and E genes	Next to patient’s head: 0/4 (0%); Coroners of rooms & anteroom: 0/12 (0%)	Patient room: 12/14 (85.7%) Patient toilet: 3/5 (60.0%) Air outlet fan: 2/3 (66.7%) Anteroom: 0/6 (0%)
Guo ZD [10]	Wuhan, China (2020)	Clinical; negative pressure wards^a^	15 patients (ICU); 24 patients (GW)	SASS 2300 Wetted Wall Cyclone Sampler: collected 9,000 L at 300 L/min (ICU & GW)	ORF 1ab and N genes	ICU (near patient): 8/18 (44.4%)^b^ VL: 1.4 copies/L ICU (near doctor office): 1/8 (12.5%)^b^ VL: 0.52 copies/L GW: 2/18 (11.1%)^b^ VL: 1.6×10^4^ copies/L	ICU (patient area): 32/54 (59.3%)^b^ ICU (air outlet): 5/14 (35.7%) GW (patient area): 6/57 (10.5%)^b^
Bae S [22]	Seoul, South Korea (2020)	Clinical; negative pressure rooms	4 patients; VL of NPS or saliva (2.59–7.68 log copies/mL)	Patients coughing 5 times onto a petri dish placed at 20 cm, with or without wearing surgical or cotton mask^c^	NM	Median VL (log copies/mL) during cough: without mask: 2.56; with surgical mask: 2.42; with cotton mask: 1.85	Outer surface of surgical and cotton mask: 8/8 (100%); 2.11 to 3.61 copies/mL Inner surface of surgical and cotton mask: 2/8 (25%); 2.00 to 3.70 copies/mL
van Doremalen N [11]	United States (2020)	Non-clinical simulation	Artificial generation of aerosol^d^	Sartorius air sampler: collected samples at 0, 30, 60, 120 and 180 min after aerosolization^e^	NM	Up to 3 hours: ↓ infectious titer (10^3.5^ to 10^2.7^ TCID_50_ per liter of air)	Plastic and stainless steel (up to 72 h);^f^ Copper < 4 hours; cardboard <24 h
Leung NHL [17]	Hong Kong, China (2020)	Clinical; at outpatient clinic; non-COVID-19 study	21 patients with common cold coronavirus^h^	G-II bioaerosol collecting device: collected exhaled breath with or without wearing surgical mask for 30 min^i^	NM	Droplet articles >5 μm: no surgical mask: 3/10 (30%); surgical mask: 0/11 (0%)^h^ Aerosol particles ≤5 μm: no surgical mask: 4/10 (40%); surgical mask: 0/11 (0%)^h^	NA

Note. ACH, air changes per hour; AIIR, airborne infection isolation room; CT, cycle threshold; GW, general ward; ICU, intensive care unit; N nucleoprotein; NA, not applicable; NM, not mentioned; NPS, nasopharyngeal swab; ORF, open reading frame; RdRp; RNA-dependent RNA polymerase; RT-PCR, reverse-transcription polymerase chain reaction; TCID_50_; 50% tissue-culture infectious dose; VL, viral load; VTM, viral transport medium.

a Isolation ward of the ICU (12 air supplies and 16 air discharges per hour) and general ward (8 air supplies and 12 air discharges per hour).

b Positive result comprising of strong and week positive by RT-PCR.

c Dacron swabs were taken samples from outer and inner surfaces of surgical mask and from the outer and inner surfaces of cotton mask.

d Aerosols (<5 μm) containing SARS-CoV-2 (10^5.25^ TCID50 per milliliter) were generated with the use of a 3-jet collison nebulizer and fed into a Goldberg drum to create an aerosolized environment. The inoculum resulted in cycle-threshold values between 20 and 22, similar to those observed in samples obtained from the upper and lower respiratory tract in humans.

e Aerosols were maintained in the Goldberg drum and samples were collected at 0, 30, 60, 120 and 180 min after aerosolization on a 47-mm gelatin filter (Sartorius). Filters were dissolved in 10 mL of DMEM containing 10% FBS, and 3 replicate experiments were performed.

f The virus titer was greatly reduced from 10^3.7^ to 10^0.6^ TCID_50_ per milliliter after 72 hours on plastic and from 10^3.7^ to 10^0.6^ TCID_50_ per milliliter after 48 hours on stainless steel.

h Not including identification of SARS-CoV-2.

i A bioaerosol collecting device, the Gesundheit-II (G-II) to capture exhaled breath particles and differentiated them into 2 size fractions, where exhaled breath coarse particles >5 μm (respiratory droplets) were collected by impaction with a 5-μm slit inertial Teflon impactor and the remaining fine particles ≤5 μm (aerosols) were collected by condensation in buffer.

We have no doubt that the air around COVID-19 patients may contain SARS-CoV-2, as extrapolated from a study on respiratory viruses including common cold–related human coronaviruses from the exhaled breath directly from the infected patients.^17^ In this study using a G-II bioaerosol collecting device, exhaled breath particles were directly captured and differentiated into 2 size fractions including respiratory droplets of particles with aerodynamic diameter >5 μm and aerosols of particles ≤5 μm. Among the exhaled air samples collected without a face mask, 6 of 10 participants (60%) with common cold coronavirus infection did not shed detectable virus in respiratory droplets or aerosols. Only 4 patients had detectable virus at low median viral load (0.3 log_10_ virus copies per sample). Assuming that the tidal volume of a young adult is 500 mL and that the average respiration rate is 16 breaths per minute, the total volume of exhaled air is 8 L per minute, which totals 240 L per 30 minutes. Given the median viral load of coronavirus of 0.3 log_10_ virus copies per each exhaled breath sample collected for 30 minutes, the concentration of viral RNA per liter of exhaled air becomes negligible (0.00125 copies per liter of air).^17^ In 2 studies (including our study) of air sampling from COVID-19 patients, the air samples were not completely composed of exhaled breath but of a mixture of both room air and exhaled air from patients.^10,12^ Therefore, negative SARS-CoV-2 RNA findings might be expected in volumes of 1,000–1,200 L of air samples in AIIRs. Even though we used an umbrella with curtain to reduce the dilution effect of air inside the AIIRs, it was not sufficient to improve the yield of SARS-CoV-2 detection.

Successful infection by SARS-CoV-2 depends on the dose of viral exposure and the host immunity. In a murine model, the infectious dose of 2003 SARS-CoV was estimated to be 43–280 plaque-forming units.^18^ Based on our experiment, 1 plaque-forming unit is equivalent to a mean of 1.67 × 10^4^ virus genome copies/mL. Assuming that the infectious dose of SARS-CoV-2 is similar to that of SARS-CoV or common cold coronaviruses, the susceptible individual may need to stay with a COVID-19 case for 19.5 years to obtain enough virus inoculum as extrapolated from the exhaled breath virus concentration detected by G-II bioaerosol collecting device. Similarly, a susceptible patient needs to stay in a Wuhan ICU for 44.5 days to become infected, theoretically (Supplementary Table 2 online), provided that the other routes of transmission are absent. In fact, prolonged exposure in the same confined environment was also required for animal-to-animal transmission, as illustrated in our caged hamster experiment.^19^ We only collected air samples from 6 patients with a total collection time of ~2 hours when they were not wearing a surgical mask; thus, we cannot completely exclude the possibility of a single erratic event of high virus inoculum being shed by the COVID-19 patients when a longer period of sampling is performed. Our previous study showed that wearing of a surgical mask by either patients or healthcare workers prevented nosocomial transmission of influenza during the pandemic of influenza A H1N1 virus in 2009.^20^ Recently, we also demonstrated that community-wide wearing of face masks could minimize the transmission of COVID-19 in Hong Kong.^21^ Our present findings may also support the usefulness of wearing surgical masks by patients in minimizing environmental contamination by their own respiratory droplets and saliva.

Although we are puzzled by the study showing that wearing surgical masks by patients infected with common cold coronaviruses could prevent their exhalation of virus-laden aerosols of particles ≤5 μm, it is possible that the filtration mechanism of a surgical mask may be sufficient prevent the transmission of such small-sized particles with coronavirus when their number is low. However, the use of a face mask to prevent dispersal of SARS-CoV-2 was challenged by a recent study on 4 patients who were instructed to cough directly onto a petri dish containing VTM, which was placed at ~20 cm from the patient’s mouth, with or without surgical or cotton mask.^22^ In this study, the shedding of virus was sporadic. In 1 patient who coughed without wearing face mask, only 1 of 2 coughing episodes yielded detectable SARS-CoV-2 RNA. Notably, the viral load in the VTM exposed to coughing from patients was highest when patient was coughing without mask (2.56 log copies/mL), followed by coughing with surgical mask (2.42 log copies/mL) and coughing with cotton mask (1.85 log copies/mL). These findings suggest that cotton masks may offer better or similar protection as surgical masks. Surprisingly, no SARS-CoV-2 RNA was detected in 3 of 4 surface swabs collected from inner sides of a surgical mask and a cotton mask, even though 3 of 4 patients produced detectable viral RNA in the petri dish placed 20 cm from patient’s mouth. Whether such apparently discrepant findings can be explained by complicated aerodynamics deserves further investigation.

Because COVID-19 is predominantly transmitted through droplet and contact routes, droplet and contact precautions should have overriding importance. The Hong Kong Special Administrative Region is one of the very few areas where community-wide wearing of face mask was practiced from the beginning of this COVID-19 epidemic.^21^ We promoted proactive infection control measures in the hospitals with enforcement of hand hygiene practice and environmental cleaning,^14,23^ and all hospitalized patients were provided with surgical masks as source control to reduce the amount of environmental contamination.^14^ However, the mandatory use of surgical masks by hospitalized patients in studies from Singapore and China was not mentioned.^10,12^ In addition, we have been promoting hand hygiene among our hospitalized patients.^24–26^ Therefore, our single-room environment with confirmed cases of COVID-19 was significantly less contaminated than that in Singapore and Wuhan, China. Of our 377 environmental samples collected before daily cleaning and disinfection, only 5% were positive for SARS-CoV-2 RNA, compared with 10%–85% in hospitals in Singapore and Wuhan, China.^10,12^ Moreover, we showed that the higher viral load of patients’ clinical samples correlated with the likelihood of environmental contamination. When the viral load of clinical samples was ≥3 log copies/mL, environmental contamination with SARS-CoV-2 could be detected. Among all sampled items and locations, patients’ mobile phones were the most frequently contaminated item. This personal electronic device is not routinely disinfected by the hospital cleaning team. This finding has implications for the disinfection policy of commonly shared electronic devices for patient care in the hospital. Ultraviolet C light-emitting disinfecting device specifically designed for electronic mobile device was advocated during this pandemic.^27,28^ The bed rail was another item highly contaminated with SARS-CoV-2 that is frequently touched by both patients and healthcare workers, as illustrated previously.^29^ Therefore, enforcement of hand hygiene in healthcare workers and patients remains the cornerstone of infection control,^24–26^ especially during the COVID-19 pandemic.^30^ Correct wearing of a surgical mask, diligent practice of hand hygiene, and thorough environmental disinfection should be sufficient for the prevention of COVID-19 in the hospital setting, except during aerosol-generating procedures or when high numbers of COVID-19 patients are concentrated in a cohort ward.

This study has several limitations. First, the patient number was low. The total process of air sampling including the preparation and instructions to patient took at least 2 hours per patient. Thus, some patients refused to join the study. Second, the temporal fluctuations of a patient’s viral load, the performance of aerosol-generating procedures, and the immunity of susceptible person were not included in the calculation of the infectious virus dose. Further investigations of the complicated aerodynamics of the SARS-CoV-2-laden aerosols, especially in the cohort ward setting or during aerosol-generating procedures, should be performed.

## Supplementary material

For supplementary material accompanying this paper visit http://dx.doi.org/10.1017/ice.2020.282. click here to view supplementary material
