# Supplementary material for: Air and environmental sampling for SARS-CoV-2 around hospitalized patients with coronavirus disease 2019 (COVID-19)
Source: Infect Control Hosp Epidemiol. 2020 Jun 8:1–8. doi: 10.1017/ice.2020.282 (PMC7327164; doi:10.1017/ice.2020.282)
Supplement: Supplementary file 1 [file S0899823X20002822sup.zip › S0899823X20002822sup004.pptx]

## Slide 1
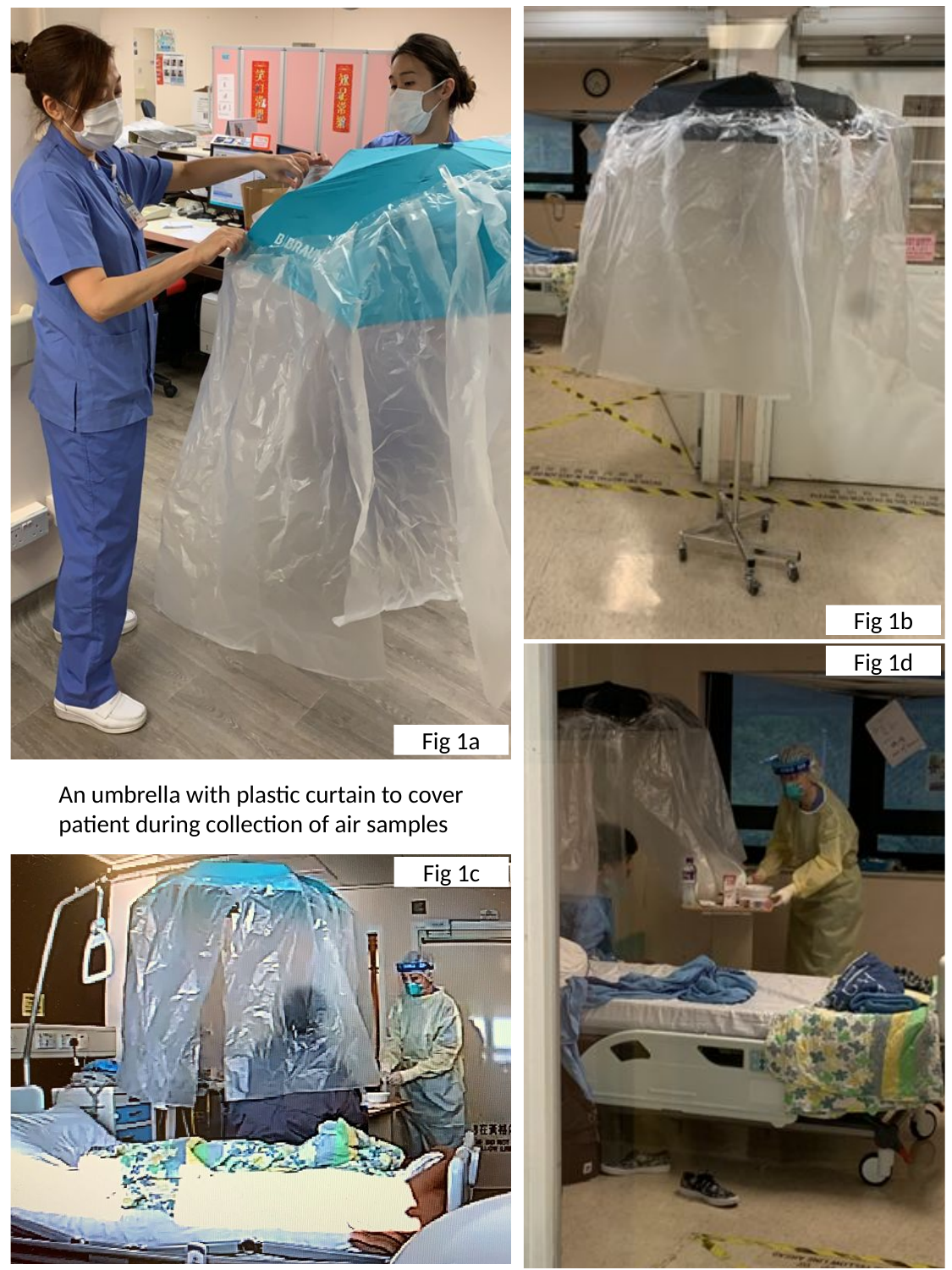

Fig 1b
Fig 1d
Fig 1a
An umbrella with plastic curtain to cover patient during collection of air samples
Fig 1c
