# Supplementary material for: Air and environmental sampling for SARS-CoV-2 around hospitalized patients with coronavirus disease 2019 (COVID-19)
Source: Infect Control Hosp Epidemiol. 2020 Jun 8:1–8. doi: 10.1017/ice.2020.282 (PMC7327164; doi:10.1017/ice.2020.282)
Supplement: Supplementary file 1 [file S0899823X20002822sup.zip › S0899823X20002822sup001.docx]

Supplementary File 1

*Viral load assay*

The viral loads of the samples were determined by the SARS-CoV-2 RdRp/Hel real-time RT-PCR as we described previously.^1^ To monitor for PCR inhibitors in the gelatin filter samples, an inhibitor control with 1 µl of SARS-CoV-2 control RNA was spiked into each additional reaction. A cycle threshold value of less than 28 was expected for the spiked control reaction. The viral loads of COVID-19 confirmed patients were determined from routine clinical samples (nasopharyngeal swab, throat swab, and deep throat saliva) on the same day of the collected air sample.

*Correlation between viral load and plaque forming unit of SARS-CoV-2*

As we did not determine the infectiousness of the air sample by cell culture, we tried to correlate the viral load results with the plaque forming units of SARS-CoV-2 in cell culture for comparison of our findings with previous reports. The same volume of virus stock harvested at 24 hours of growth from infected Vero E6 cell line was simultaneously assayed for viral load and plaque forming units. Briefly, Vero E6 cells were maintained in Dulbecco’s Modified Eagle’s Medium (DMEM) with 10% fetal bovine serum (FBS), 1% penicillin, and 1% streptomycin. The cells were seeded one day before the experiment at 2×10^5^ cells per well in 12-well plates and were allowed to grow at 37^o^C overnight. For the plaque assay, serially diluted samples were inoculated on the cells for 1 hour. Afterwards, 3% agarose/phosphate buffered saline (PBS) was mixed with DMEM with 1.5% FBS at a 1:2 ratio and applied onto the cells. The cells were incubated for 96 hours for plaque formation. The plaques were visualized by staining the plates with 1% crystal violet in 20% ethanol/distilled water for 15 minutes.

Reference:

1. Chan JF, Yip CC, To KK, Tang TH, Wong SC, Leung KH, et al. Improved molecular diagnosis of COVID-19 by the novel, highly sensitive and specific COVID-19-RdRp/Hel real-time reverse transcription-polymerase chain reaction assay validated in vitro and with clinical specimens. J Clin Microbiol. 2020;58:e00310-20.
