# Supplementary material for: Air and environmental sampling for SARS-CoV-2 around hospitalized patients with coronavirus disease 2019 (COVID-19)
Source: Infect Control Hosp Epidemiol. 2020 Jun 8:1–8. doi: 10.1017/ice.2020.282 (PMC7327164; doi:10.1017/ice.2020.282)
Supplement: Supplementary file 1 [file S0899823X20002822sup.zip › S0899823X20002822sup003.docx]

Supplementary Table 2. Correlation between inhalation of contaminated air and infection of SARS-CoV-2

|  | Settings and parameters | Value |
| --- | --- | --- |
| I. | In-vitro virological experiment |  |
| (A) | Infectious dose (plaque forming unit) ^a^ | 43 |
| (B) | Viral load (copies / ml) per 1 plaque forming unit ^b^ | 1.67 x 10^4^ |
| (C) | Infectious dose (viral copies / ml) [= (A) x (B)] | 7.18 x 10^5^ |
|  |  |  |
| II. | Human respiratory physiology |  |
| (D) | Tidal volume (liter) per breath | 0.5 |
| (E) | Respiratory rate for an adult per minute ^c^ | 16 |
| (F) | Volume of exhaled air (liter) per minute [= (D) x (E)] | 8 |
|  |  |  |
| Ⅲ. | Breathing experiment for human coronavirus [1] ^d^ |  |
| (G) | Median viral copies in aerosol faction of exhaled breath for 30 minutes (without wearing a face mask) | 2 |
| (H) | Median viral copies in aerosol faction of exhaled breath per minutes (without wearing a face mask) [= (G) / 30] | 0.07 |
|  |  |  |
| IV. | Conclusion (from I & II & III) |  |
| (I) | Time (year) of inhalation of aerosol fraction required to reach infectious dose [= (C) / (H) / 60 minutes / 24 hours / 365 days] ^e^ | 19.5 |
|  |  |  |
| Ⅴ. | Air sampling collection in an ICU in Wuhan, China [2] |  |
| (I) | Viral copies per liter of air sample | 1.4 |
| (J) | Viral copies in 9000 liters during 30 minutes of air collection [= (I) x 9000] | 12,600 |
| (K) | Viral copies per minute of air collection [= (J) / 30] | 420 |
|  |  |  |
| VI. | Conclusion (from I & II & V) |  |
| (L) | Volume of air (liter) inhaled in ICU required to reach infectious dose [= (C) / (I)] | 512,857 |
| (M) | Time (day) of inhalation of air sample in ICU required to reach infectious dose [= (L) / (F) / 60 minutes / 24 hours] ^f^ | 44.5 |

Note. ICU, intensive care unit.

^a^ The dose of SARS-CoV corresponding to 10% responses (illness) was used for the calculation [1].

^b^ The mean value of viral load illustrated in Supplementary Table 1 was used.

^c^ Respiratory rate of an adult ranged from 12 to 20 per minute. A value of 16 was chosen for calculation.

^d^ Assuming of exhalation of human coronavirus is similar to SARS-CoV-2 in an adult. The value of viral copies is expressed in numeric instead of log scale as reported in the reference [3].

^e^ Assuming the unit of ml of fluid and ml of air is equivalent, and the susceptible person is inhaling the air from the infected person with the exhaled air only constituted of aerosol.

^f^ The actual copies of SARS-CoV-2 may be affected by the rate of air sampling. The lower the rate of air sampling, the longer the time required to collect 9000 liters of air in intensive care unit. The time factor may affect the amount of SARS-CoV-2 generated from the clinical activities in intensive care unit.

median viral loads after coughs without a mask, with a surgical mask, and with a cotton mask were 2.56 log copies/mL, 2.42 log copies/mL, and 1.85 log copies/mL,

Reference

1. Leung NHL, Chu DKW, Shiu EYC, Chan KH, McDevitt JJ, Hau BJP, et al. Respiratory virus shedding in exhaled breath and efficacy of face masks. Nat Med. 2020;26:676‐680.
2. Guo ZD, Wang ZY, Zhang SF, Li X, Li L, Li C, et al. Aerosol and Surface Distribution of Severe Acute Respiratory Syndrome Coronavirus 2 in Hospital Wards, Wuhan, China, 2020. Emerg Infect Dis. 2020;26:10.3201/eid2607.200885.
3. Watanabe T, Bartrand TA, Weir MH, Omura T, Haas CN. Development of a dose-response model for SARS coronavirus. Risk Anal. 2010;30:1129-38.
