# Supplementary material for: Air and environmental sampling for SARS-CoV-2 around hospitalized patients with coronavirus disease 2019 (COVID-19)
Source: Infect Control Hosp Epidemiol. 2020 Jun 8:1–8. doi: 10.1017/ice.2020.282 (PMC7327164; doi:10.1017/ice.2020.282)
Supplement: Supplementary file 1 [file S0899823X20002822sup.zip › S0899823X20002822sup002.docx]

Supplementary Table 1. The correlation between viral load and plaque forming units performed in triplicate

| Replicate  number | Viral load (RdRP gene copies/ml) | Plaque forming unit (PFU) | Viral load (copies/ml) per 1 PFU | Remark |
| --- | --- | --- | --- | --- |
| 1 | 2.47 x 10^8^ | 1.83 x 10^4^ | 1.35 x 10^4^ |  |
| 2 | 2.37 x 10^8^ | 1.03 x 10^4^ | 2.30 x 10^4^ |  |
| 3 | 1.62 x 10^8^ | 1.18 x 10^4^ | 1.37 x 10^4^ |  |
|  |  |  | 1.67 x 10^4^ (± 4.43 x 10^3^) | Mean (± SD) |

Note. RdRP, RNA-dependent RNA polymerase
